# Accuracy of imputation to whole-genome sequence data in Holstein Friesian cattle

**DOI:** 10.1186/1297-9686-46-41

**Published:** 2014-07-15

**Authors:** Rianne van Binsbergen, Marco CAM Bink, Mario PL Calus, Fred A van Eeuwijk, Ben J Hayes, Ina Hulsegge, Roel F Veerkamp

**Affiliations:** 1 Animal Breeding and Genomics Centre, Wageningen UR Livestock Research, P.O. Box 338, 6700 AH Wageningen, the Netherlands; 2 Biometris, Wageningen University and Research Centre, P.O. Box 100, 6700 AC Wageningen, the Netherlands; 3 Biosciences Research Division, Department of Environment and Primary Industries, 1 Park Drive, Bundoora 3083, Australia

## Abstract

**Background:**

The use of whole-genome sequence data can lead to higher accuracy in genome-wide association studies and genomic predictions. However, to benefit from whole-genome sequence data, a large dataset of sequenced individuals is needed. Imputation from SNP panels, such as the Illumina BovineSNP50 BeadChip and Illumina BovineHD BeadChip, to whole-genome sequence data is an attractive and less expensive approach to obtain whole-genome sequence genotypes for a large number of individuals than sequencing all individuals. Our objective was to investigate accuracy of imputation from lower density SNP panels to whole-genome sequence data in a typical dataset for cattle.

**Methods:**

Whole-genome sequence data of chromosome 1 (1737 471 SNPs) for 114 Holstein Friesian bulls were used. Beagle software was used for imputation from the BovineSNP50 (3132 SNPs) and BovineHD (40 492 SNPs) beadchips. Accuracy was calculated as the correlation between observed and imputed genotypes and assessed by five-fold cross-validation. Three scenarios S40, S60 and S80 with respectively 40%, 60%, and 80% of the individuals as reference individuals were investigated.

**Results:**

Mean accuracies of imputation per SNP from the BovineHD panel to sequence data and from the BovineSNP50 panel to sequence data for scenarios S40 and S80 ranged from 0.77 to 0.83 and from 0.37 to 0.46, respectively. Stepwise imputation from the BovineSNP50 to BovineHD panel and then to sequence data for scenario S40 improved accuracy per SNP to 0.65 but it varied considerably between SNPs.

**Conclusions:**

Accuracy of imputation to whole-genome sequence data was generally high for imputation from the BovineHD beadchip, but was low from the BovineSNP50 beadchip. Stepwise imputation from the BovineSNP50 to the BovineHD beadchip and then to sequence data substantially improved accuracy of imputation. SNPs with a low minor allele frequency were more difficult to impute correctly and the reliability of imputation varied more. Linkage disequilibrium between an imputed SNP and the SNP on the lower density panel, minor allele frequency of the imputed SNP and size of the reference group affected imputation reliability.

## Background

One advantage of using whole-genome sequence data over genotypes from SNP (single nucleotide polymorphisms) panels for genome-wide association studies (GWAS) and genomic prediction is that polymorphisms causing genetic differences can be included in whole-genome sequence data. Because the causative mutation is included, decay in linkage disequilibrium (LD) between a SNP and the causative mutation by recombination events is not an issue. Accordingly, testing variants directly associated with a given trait is possible and may lead to higher accuracy in GWAS and genomic predictions. Moreover, since there is no decay in LD when using sequence data compared to traditional smaller-sized marker panels, genomic selection across generations and across breeds may be improved e.g. [1-3].

Costs to generate whole-genome sequence data are decreasing rapidly. It is expected that, in the next few years, whole-genome sequence data will be widely available for crops and livestock, as is already the case for human studies [4]. Despite the fact that costs of sequencing are decreasing, it is still expensive to sequence large numbers of individuals. A less expensive approach to produce sequence genotypes for a large number of individuals is to impute from lower density marker panels to whole-genome sequence data. In this case, a core set of individuals is fully sequenced, and the lower density genotypes of the remaining individuals will be imputed to whole-genome sequence genotypes using the sequenced individuals as reference [5-8].

However, using sequence data may not lead to higher accuracy in genomic predictions and GWAS if the accuracy of imputation to sequence data is too low. Accuracy of imputation was studied in barley with 3200 SNPs [9], in maize with 35 000 SNPs [10], in sheep with 50 000 SNPs [11] and in cattle with 50 000 SNPs e.g. [12] and 777 000 SNPs e.g. [13], among others. The general tendency in those studies was that the accuracy of imputation increased with an increasing number of SNPs on the lower density marker panel, a decreasing distance between the imputed SNP and the nearest SNP on the lower density marker panel, an increasing minor allele frequency (MAF) of imputed SNPs, an increasing level of LD (linkage disequilibrium), and an increasing number of close relatives between imputed and reference individuals. In all those studies, imputation was done from low-density panels to higher density panels but not to whole-genome sequence data.

In contrast to crops and livestock, human sequence data are available and accuracy of imputation to sequence data has been investigated e.g. [14-16], which showed that accuracy of imputation was influenced by reference group composition (e.g. size or populations included), number of markers on the lower density marker panel, and MAF of imputed SNPs. Moreover, according to Li *et al.*[16], these factors influenced accuracy of imputation especially in the case of SNPs with a MAF below 0.05. For imputation of SNPs with a MAF below 0.005, it was necessary that the reference group included at least 1200 individuals and for imputation of SNPs with a MAF between 0.005 and 0.05, only about 40% of the SNP genotypes were imputed with 1200 individuals in the reference group.

Crop and livestock populations differ from human populations, in extent of LD and population structure [17-19]. In cattle, effective population size of some individual breeds has decreased rapidly to about 100 due to intense selection [19-21]. Consequently, LD in cattle breeds extends on relatively long distances. This is also true for many other domestic animal and plant populations (e.g. dogs or barley), but not for human populations [17,18]. When using whole-genome sequence data, differences in extent of LD and population structure may affect imputation accuracies more in crop or livestock analyses than in human analyses.

The objective of this study was to investigate the accuracy of imputation of genotypes from SNP panels to whole-genome sequence data in a typical dataset of domestic animals and to gain insights on the factors that affect accuracy of imputation, such as number of sequenced individuals, number of SNPs on the lower density marker panel, location and MAF of the imputed SNPs. Because in practice true genotypes are unknown, it is important to understand the underlying factors that influence imputation accuracy. Holstein Friesian cattle data provided by the 1000 bull genomes project [22,23] was used in this study.

## Methods

### Genotypic data

Whole-genome sequence data of 114 Holstein Friesian bulls were provided by the 1000 bull genomes project (Run 2.0) [22,23]. Bulls that originated from Australia, Canada, Denmark, Finland, France, Germany, Sweden, The Netherlands, UK, and USA, were identified as key ancestors of the global Holstein Friesian population. Each bull was sequenced using Illumina HiSeq Systems (Illumina Inc., San Diego, CA). Alignment, variant calling, and quality controls were done in a multi-breed population with sequenced Holstein Friesian, Fleckvieh, Jersey, and Angus bulls as described by Daetwyler *et al.*[22]. Variants used in our study were SNPs and INDELs (both considered as SNPs here). Two alleles (A and B) per SNP were assumed with a value of 0, 1, or 2 for genotype AA, AB, or BB, respectively. To save computing time and space, only SNPs on *Bos taurus* autosome 1 (BTA1) were used. Similar results were expected for other chromosomes.

A set of sequence variants and genotypes that can be used to test imputation programs is available at request via http://www.1000bullgenomes.com[23].

### Imputation

Beagle 3.3.2 software [5] with default parameter settings was used for imputation. No SNP edits were performed prior imputation. For each individual, the most likely genotypes were used and they were assumed to be unphased, for both the reference and validation sets. Moreover, it was assumed that all individuals were unrelated. Accuracy of imputation (*r*) was calculated as the correlation between observed and imputed genotypes. Imputed genotypes were assessed by estimated *B*-allele dosage, which had a value between 0 and 2 and was calculated using posterior genotype probabilities as estimated by Beagle: 0 * P(AA) + 1 * P(AB) + 2 * P(BB). SNPs with fixed observed genotypes or estimated *B*-allele dosages for one or more validation groups were removed. Accuracy of imputation ranged between -1 (opposite genotype imputed) and +1 (correct genotype imputed). An imputation accuracy with a value around 0 meant random imputation.

To assess imputation accuracy, five-fold cross validation was performed. Individuals were randomly divided in five groups, group 1 to 5, and each group was used as validation set once. For validation individuals, SNP genotypes for SNPs corresponding to the Illumina BovineSNP50 BeadChip (Illumina Inc., San Diego, CA; 54 609 SNPs) or Illumina BovineHD BeadChip (Illumina Inc., San Diego, CA; 777 962 SNPs) were retained, while the remaining SNPs on the sequence panel were masked.

### Scenarios

To study the effect of number of sequenced individuals on imputation accuracy, three scenarios were considered: S80, S60, and S40. Reference group in scenarios S80, S60 and S40 contained 80% (all, except validation individuals), 60% and 40% of the individuals, respectively. In scenarios S40 and S60, the two or three following groups were designated as reference group. For example for scenario S60, if individuals in group 1 were designated as validation individuals, then individuals in group 2, 3, and 4 were designated as reference individuals.

According to VanRaden *et al.*[13], accuracy of imputation from 3 K and 6 K panels to the BovineHD beadchip was improved if the genotypes were imputed first to the BovineSNP50 and then to the BovineHD beadchip instead of directly to the BovineHD beadchip. To study if this stepwise imputation approach also improved accuracy of imputation from the BovineSNP50 beadchip to whole-genome sequence data, a stepwise imputation was studied in scenario S40. Individuals in the two following groups were reference individuals for imputation to the BovineHD beadchip (step 1) and individuals in the two previous groups were reference individuals for imputation to whole-genome sequence data (step 2). For example, if individuals in group 2 were designated as validation individuals, then individuals in group 3 and 4 were assigned to the reference group for step 1, and individuals in group 5 and 1 were assigned to the reference group for step 2.

### Factors that affect imputation accuracy

Factors that can influence imputation accuracy per SNP are number of sequenced individuals, distance (in base pairs) and MAF difference between an imputed SNP and its nearest SNP on the lower density marker panel, and MAF of imputed SNPs. MAF was calculated for each SNP based on all 114 individuals. For graphical representation and to illustrate the average behavior of SNPs, SNPs were binned in groups of 1000 based on distance or MAF (difference), and these binned SNPs were used to study imputation reliability (*r*^2^).

To investigate the relationship between imputation reliability for a SNP and the factors that may influence its value, a few simple functions were used. Although haplotypes (and not single SNPs) are used for imputation of missing SNPs, our first assumption was that imputation reliability is based on LD between known and unknown SNPs, and our second assumption was that MAF together with number of sequenced individuals will affect imputation reliability.

Two functions were used to model LD between two SNPs: one was based on distance [24] and one was based on difference in MAF [25]. The first function describes LD decay (*r*_*dist*_^2^) based on effective population size (*Ne*) and distance of an imputed SNP to its nearest SNP on the lower density marker panel (*c*; in Morgan):

$$r_{\text{dist}}^{2}=\frac{1}{4*\mathrm{Ne}*c+1}.$$

*Ne* was assumed to be equal to 100 or 1000 and for distances, it was assumed that 10^6^ base-pairs (1 Mb) are equal to 1 centiMorgan (cM) [26,27]. The second function describes the general upper limit for LD $\left(r_{\text{dMAF}}^{2}\right)$ based on difference in MAF between an imputed SNP and its nearest SNP on the lower density marker panel (dMAF) [25]:

$$r_{\mathrm{dMAF}}^{2}=\frac{1-4\text{dMAF}}{2\text{dMAF}+1}.$$

If two SNPs differ in MAF, LD between those SNPs is expected to be low [28,29].

These two functions do not account for the MAF of imputed SNPs or number of reference individuals. With a low number of reference individuals, the probability that individuals carry the rare allele of a SNP with a low MAF is lower, thus increasing the number of reference individuals may increase imputation reliability of this SNP. To our knowledge, there is no theoretical function that describes the relationship between imputation reliability or LD and MAF of imputed SNPs or number of reference individuals. Therefore, an empirical function was derived by fitting a Michaelis-Menten function [30] on the data:

$$r_{\text{MAF}}^{2}=\frac{V_{\text{max}}*\text{MAF}}{K_{m}+\mathrm{MAF}},$$

where $r_{\text{MAF}}^{2}$ is the imputation reliability, *V*_*max*_ is the estimate of the upper limit of $r_{\text{MAF}}^{2}$ and *K*_*m*_ is the deflection point, i.e. the estimated MAF when $r_{\mathrm{MAF}}^{2}$ = 1/2*V*_*max*_. The Michaelis-Menten function is often used in studies on enzyme kinetics that describe the rate of enzymatic reactions based on substrate concentration [30]. This function was chosen because of its simplicity (two meaningful parameters) and its agreement with the observed data (starting from 0, it increases rapidly at the beginning and asymptotically approaches its maximum).

The three functions mentioned each explain a part of the imputation reliability. For overall imputation reliability $\left(r_{\text{total}}^{2}\right)$ the functions were multiplied:

$$r_{\text{total}}^{2}=r_{\text{dist}}^{2}*r_{\text{dMAF}}^{2}*r_{\text{MAF}}^{2}.$$

In the functions for $r_{\text{dist}}^{2}$ and $r_{\text{dMAF}}^{2}$, the nearest SNP on the lower density marker panel was used although it may not be the SNP that has the highest LD with the imputed SNP. To take this into account, for each SNP, $r_{\text{dist}}^{2}*r_{\text{dMAF}}^{2}$ was estimated for the five nearest SNPs on the lower density marker panel and, for each imputed SNP, SNPs on the lower density marker panel that had the highest value for $r_{\text{dist}}^{2}*r_{\text{dMAF}}^{2}$ were selected. Next, the parameters *V*_*max*_ and *K*_*m*_ were estimated by fitting $r_{\text{MAF}}^{2}$. Finally, $r_{\text{total}}^{2}$ was calculated and imputed SNPs were grouped with 1000 SNPs into bins with similar values of $r_{\text{total}}^{2}$ and plotted against the observed *r*^2^ from the sequence data.

## Results

### Whole-genome sequence data

**Figure 1:**
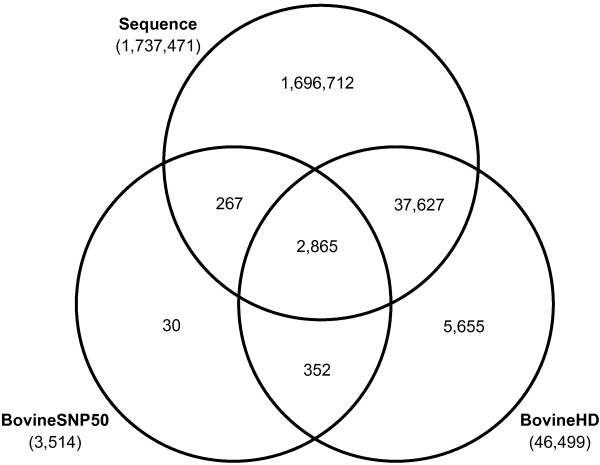
**Number of SNPs on BTA 1.** Venn diagram showing number of SNPs on BTA1 in the two lower density marker panels (BovineSNP50 and BovineHD) and in whole-genome sequence data and overlapping numbers.

BTA1 is the largest bovine chromosome and contains approximately 160.10^6^ bp. In the current 1000 bull genomes dataset, 1737 471 SNPs (of which 5.5% were INDELs) were called on BTA1 based on a multi-breed population. Of these SNPs, 76.8% showed variation within the 114 Holstein Friesians. The BovineSNP50 and BovineHD panels contained respectively 3514 and 46 499 SNPs on BTA1, however, not all these SNPs were found in the sequence data. For the BovineSNP50 panel, 3132 SNPs (0.18% of the SNPs in the sequence data) and the BovineHD panel, 40 492 SNPs (2.33% of the SNPs in the sequence data) were found in the sequence data. Figure 1 presents a Venn diagram of the numbers of SNPs on BTA1 in the two lower density marker panels and in the whole-genome sequence data and numbers of overlapping SNPs.

### Accuracy of imputation

Mean accuracy of imputation per SNP was assessed by cross-validation. For imputation from the BovineSNP50 beadchip to sequence data, it ranged between 0.37 for scenario S40 and 0.46 for S80, and for imputation from the BovineHD beadchip to sequence data, it ranged between 0.77 for scenario S40 to 0.83 for S80 (Table 1). Standard deviations ranged from 0.36 to 0.37 for imputation from the BovineSNP50 beadchip, and from 0.27 to 0.29 for imputation from the BovineHD beadchip. In comparison to direct imputation from the BovineSNP50 beadchip to sequence data, stepwise imputation from the BovineSNP50 to the BovineHD beadchip and then to sequence data improved accuracy per SNP from 0.28 to 0.65 for scenario S40. However, it was still lower than the accuracy of imputation from the BovineHD panel to sequence data (0.77). Accuracy per SNP for stepwise imputation was found to be similar to the product of imputation accuracies for the two steps.

**Table 1:** Mean accuracy of imputation per SNP

		**Mean**	**SD**	**Minimum**	**Maximum**	**Nb SNPs**
**S80**	**BovineHD**	0.83	0.27	-0.43	1.00	744 896
	**BovineSNP50**	0.46	0.37	-0.54	1.00	768 907
**S60**	**BovineHD**	0.81	0.27	-0.37	1.00	736 216
	**BovineSNP50**	0.43	0.36	-0.58	1.00	780 388
**S40**	**BovineHD**	0.77	0.29	-0.33	1.00	739 859
	**BovineSNP50**	0.37	0.36	-0.40	1.00	764 439
**2-step**	**Step 1**	0.83	0.15	-0.17	1.00	32 880
	**Step 2**	0.77	0.29	-0.33	1.00	739 859
	**Overall**	0.65	0.30	-0.41	1.00	764 912

Mean, standard deviation (SD), minimum and maximum accuracy of imputation per SNP on BTA1 for different combinations of scenarios and lower density marker panels; for scenario S40, accuracy of stepwise imputation is also shown for step 1 (BovineSNP50 to BovineHD), step 2 (BovineHD to sequence), and overall; number of SNPs used for analyses are presented in the last column.

Mean accuracy of imputation per individual was higher than mean accuracy per SNP. For imputation from the BovineSNP50 panel and from the BovineHD panel to sequence data, mean accuracies ranged from 0.78 for scenario S40 to 0.95 for S80, and from 0.93 for scenario S40 to 0.95 for S80, respectively (Table 2). Reasons for this difference are discussed below. For imputation from either of the lower density marker panels, standard deviation was 0.04 for all scenarios. As for accuracy per SNP, imputation accuracy per individual was improved with stepwise imputation from the BovineSNP50 beadchip to sequence data for scenario S40 and reached a value similar to the product of imputation accuracies of each step.

**Table 2:** Mean accuracy of imputation per individual

		**Mean**	**SD**	**Min**	**Max**	**Nb SNPs**
**S80**	**BovineHD**	0.95	0.04	0.70	0.97	744 896
	**BovineSNP50**	0.80	0.04	0.61	0.85	768 907
**S60**	**BovineHD**	0.94	0.04	0.70	0.97	736 216
	**BovineSNP50**	0.79	0.04	0.61	0.85	780 388
**S40**	**BovineHD**	0.93	0.04	0.69	0.96	739 859
	**BovineSNP50**	0.78	0.04	0.60	0.85	764 439
**2-step**	**Step 1**	0.92	0.07	0.53	0.99	32 880
	**Step 2**	0.93	0.04	0.69	0.96	739 859
	**Overall**	0.86	0.07	0.53	0.95	764 912

Mean, standard deviation (SD), minimum and maximum accuracy of imputation per individual on BTA1 for different combinations of scenarios and lower density marker panels; for scenario S40, accuracy of stepwise imputation is also shown for step 1 (BovineSNP50 to BovineHD), step 2 (BovineHD to sequence), and overall; number of SNPs used for analyses are presented in the last column.

### Factors that influence imputation accuracy

**Figure 2:**
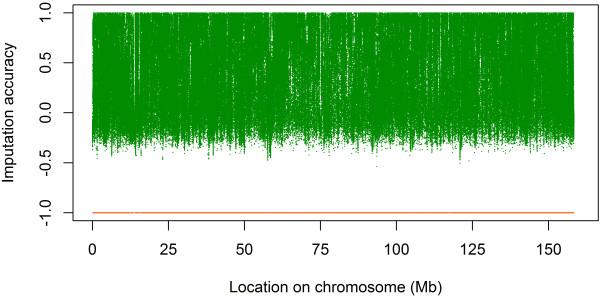
**Accuracy of imputation from the BovineSNP50 beadchip on BTA1.** Location on BTA1 versus accuracy of imputation from the BovineSNP50 beadchip to whole-genome sequence data for scenario S80; each green dot represents a SNP; orange dots at -1 are locations of SNPs of the BovineSNP50 beadchip.

**Figure 3:**
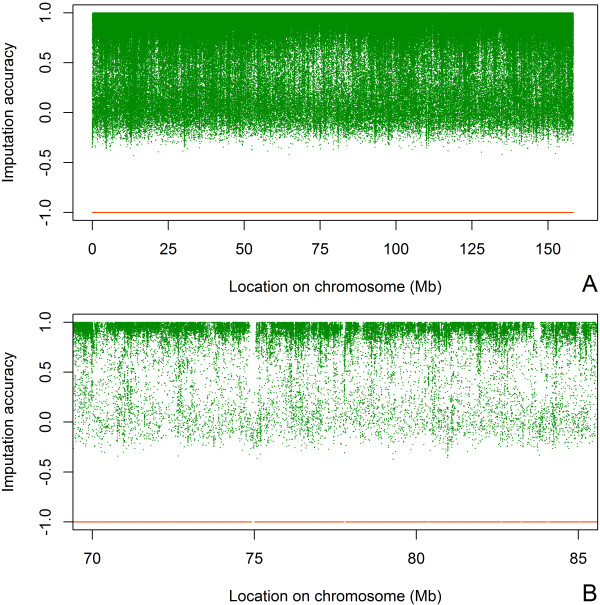
**Accuracy of imputation from the BovineHD beadchip on BTA1. (A)** for the complete BTA1. **(B)** for the region between 70 and 85 Mb on BTA1. Location on BTA1 versus accuracy of imputation from the BovineHD beadchip to whole-genome sequence data for scenario S80; each green dot represents a SNP; orange dots at -1 are locations of SNPs of the BovineHD beadchip.

The range of variation for imputation accuracies per SNP was large (Table 1). In Figures 2 and 3, this variation is illustrated for all SNPs on BTA1 for scenario S80. More SNPs had an accuracy above 0.5 for imputation from the BovineHD than from the BovineSNP50 beadchip. However, even with imputation from the BovineHD panel, SNPs from some regions of the genome were still imputed with low accuracy. For example, around the position 75.10^3^ Mb there is a region in which the distance between imputed SNPs and SNPs on the BovineHD panel is large and for which imputation was difficult (Figure 3B). This region contained SNPs that are on the BovineHD panel, but since they did not segregate in the sequence data, no genotypes were available.

**Figure 4:**
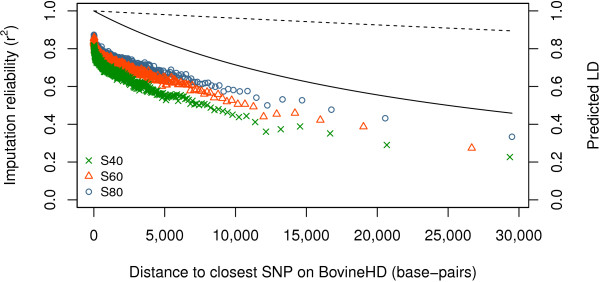
**Distance to the nearest SNP on the BovineHD beadchip versus mean imputation reliability.** Distance to the nearest SNP on the BovineHD beadchip versus mean imputation reliability for imputation from the BovineHD panel to whole-genome sequence data on BTA1 for the three scenarios (S40, S60, and S80); SNPs were grouped in bins of 1000 SNPs with similar distance; the predicted LD $\left(r_{\text{dist}}^{2}\right)$ was calculated with assumed effective population sizes (*Ne*) of 100 (dashed line) and 1000 (solid line).

Figure 4 shows the mean imputation reliability versus distance to the nearest SNP on the BovineHD beadchip for the three scenarios. Imputation reliability (imputation accuracy squared) decreased with increasing distance between imputed SNP and nearest SNP on the BovineHD panel. This decrease in imputation reliability follows the decay in LD, described as $r_{\text{dist}}^{2}$, for *Ne* = 1000. Even at very small distances, the observed imputation reliability is lower than $r_{\text{dist}}^{2}$. In addition to this distance effect, reference group size has an effect. Since imputations from the BovineHD and BovineSNP50 panels showed similar patterns for distance and all other factors, only the results for the imputation from the BovineHD panel are shown.

**Figure 5:**
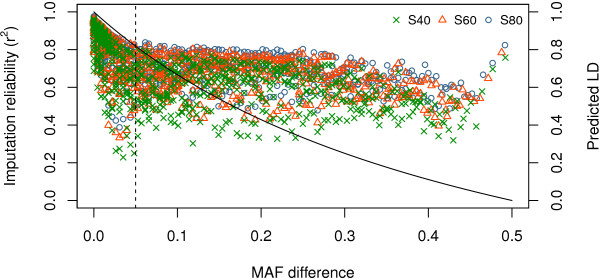
**Differences in MAF with the nearest SNP on the BovineHD beadchip versus mean imputation reliability.** Differences in MAF between imputed SNP and the nearest SNP on the BovineHD beadchip versus predicted LD $\left(r_{\text{dMAF}}^{2}\right)$ and versus mean imputation reliability for imputation from the BovineHD panel to whole-genome sequence data on BTA1 for the three scenarios (S40, S60, and S80); SNPs were grouped in bins of 1000 SNPs with similar MAF differences.

The difference in MAF between imputed SNPs and their nearest SNPs on the BovineHD beadchip determines the maximum LD between two SNPs. Figure 5 shows this MAF difference versus $r_{\text{dMAF}}^{2}$ and versus mean imputation reliability for imputation from the BovineHD beadchip for all three scenarios. For differences in MAF below 0.05, imputation reliability was below $r_{\text{dMAF}}^{2}$, which was in agreement with expectation based on maximum LD. For larger differences in MAF, observed imputation reliabilities were above estimations from $r_{\text{dMAF}}^{2}$. This pattern implies that other SNPs than only the nearest SNP on the BovineHD panel influenced imputation reliability.

**Figure 6:**
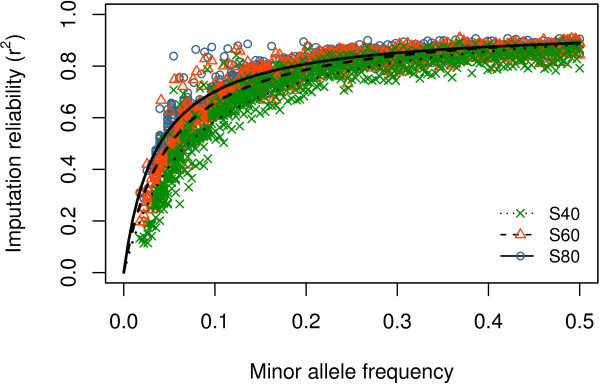
**Effect of MAF of imputed SNP and number of reference individuals on reliability of imputation.** Combined effect of MAF of imputed SNPs and scenario (S40, S60, and S80) on reliability of imputation from the BovineHD beadchip to whole-genome sequence data on BTA1; SNPs per scenario were grouped in bins of 1000 SNPs with similar MAF; for each scenario a Michaelis-Menten function was fitted.

The effect of MAF of imputed SNPs on imputation reliability is shown in Figure 6, with a Michaelis-Menten curve fitted for each scenario separately. Imputation reliability increased with increasing MAF. This increase in imputation reliability was more pronounced at a MAF below 0.2. The estimated value for the upper limit of $r_{\text{MAF}}^{2}$ (*V*_*max*_) was 1.01 (SE = 0.007) for scenario S40, 0.98 for S60 (SE = 0.005), and 0.95 (SE = 0.004) for S80. The maximum value of $r_{\text{MAF}}^{2}$ at the maximum MAF value (MAF = 0.5) was 0.881 for scenario S40, 0.893 for S60, and 0.886 for S80. The estimated MAF when $r_{\text{MAF}}^{2}$ = 1/2*V*_*max*_, or at the deflection point *K*_*m*_ was equal to 0.073 (SE = 0.002) for scenario S40, 0.049 (SE = 0.001) for S60 and 0.036 (SE = 0.001) for S80.

**Figure 7:**
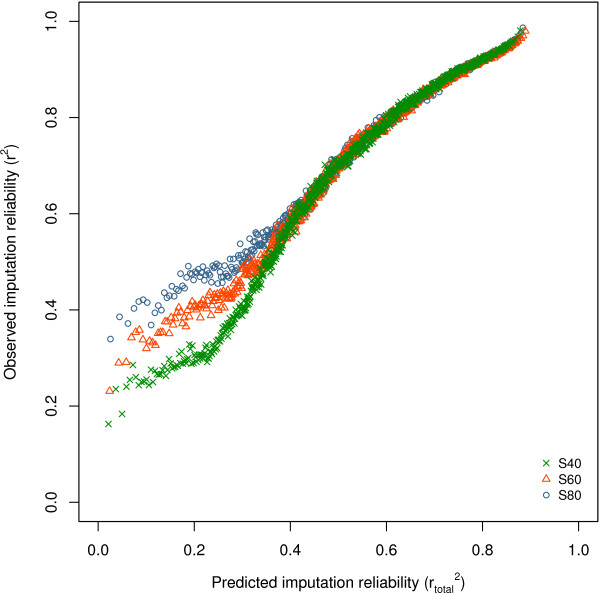
**Overall prediction of imputation reliability versus observed imputation reliability.** Overall prediction of imputation reliability ($r_{\text{total}}^{2}$, *Ne* = 1000) plotted against observed imputation reliability for imputation from the BovineHD panel to whole-genome sequence data on BTA1 for three scenarios (S40, S60, and S80); SNPs were grouped in bins of 1000 SNPs with similar $r_{\text{total}}^{2}$.

Figure 7 shows the overall estimation of imputation reliability ($r_{\text{total}}^{2}$, *Ne* = 1000) against observed imputation reliability for the three scenarios (S40, S60, S80). The estimated $r_{\text{total}}^{2}$ followed the observed reliabilities closely, although the estimated $r_{\text{total}}^{2}$ were higher than the observed reliabilities. At low $r_{\text{total}}^{2}$ , the observed imputation reliability deviated more from estimated $r_{\text{total}}^{2}$. In particular, scenarios with a higher number of individuals showed larger observed imputation reliabilities compared to the estimated $r_{\text{total}}^{2}$.

## Discussion

### Imputation from the lower density panel

Our objective was to investigate accuracy of imputation from the lower density SNP panels to whole-genome sequence data in Holstein Friesian cattle. Accuracy of imputation was defined as the correlation between observed genotypes and the imputed *B*-allele dosages. Mean accuracy of imputation per SNP to whole-genome sequence data was equal to 0.46 with 0.18% of SNPs known (BovineSNP50), and 0.83 with 2.33% of SNPs known (BovineHD). We chose to use the correlation between observed and imputed genotypes to measure accuracy of imputation, whereas most studies used percentage of correctly imputed SNPs. Compared to correlation between observed and imputed genotypes, percentage of correctly imputed SNPs does not account for the (low) MAF of imputed SNPs. A necessary condition for correlation between two random variables is that both variables show variation. Therefore, SNPs with fixed observed genotypes or estimated *B*-allele dosages for one or more validation groups were removed. This might have caused a positive bias in the results, because of removal of monomorphic loci with poor imputation. In other studies e.g. [11,13,31], criteria such as MAF greater than 0.01 were used in data editing procedures. If this type of criteria had been applied to the sequence data in our study, a large number of SNPs (987 514) would have been removed, which is similar to what occurred with the criterion chosen here.

Previous studies showed that increasing the number of close relatives between imputed and reference individuals increased imputation accuracy [9-11,32]. The sequenced bulls in this study were key ancestors of the global Holstein Friesian population and in general, were not very closely related. In fact, in some cases, they were chosen to be as little related as possible, in order to maximize sequencing effort of unique chromosome segments. A genomic relationship matrix [33] was constructed based on SNPs found on BTA1. About 90% of the off-diagonals were below 0.125 and 0.5% were above 0.5 (results not shown). In practice, these sequenced bulls will be used as reference individuals to impute genotypes of other individuals in the current population, which might be their progeny or otherwise closely related individuals. Therefore, it is expected that, in practice, imputation accuracies will be higher than those estimated in this study.

SNPs used in this study were called in a larger multi-breed population than the 114 Holstein individuals included here. Ideally, to better mimic the reality and answer the question on how many individuals need to be sequenced, the number of reference individuals used in the three scenarios should also be used for variant calling. This is important since the set of individuals used for variant calling influences the called genotypes and therefore a bias might be introduced in this study. However, we expect that the effect on the results is small, because we disregarded SNPs that did not show variation in either the reference or validation set. These are also SNPs that will not be called if only the Holstein individuals are used for variant calling. Another deviation from a real situation is that, for imputation, we assumed that the called genotypes from the sequence data were true genotypes, while it would have been more correct to use the probabilities of inferred genotypes from the sequence data as starting point for imputation. Therefore, imputation accuracies estimated in this study may differ slightly from accuracies obtained from “true genotypes”.

Mean imputation accuracy per SNP from the BovineSNP50 panel to whole-genome sequence data was below 0.46. Our results showed that an alternative approach, i.e. using stepwise imputation from the BovineSNP50 to the BovineHD panel and then to sequence data, also yielded high accuracies of imputation. For example, in scenario S40, accuracy of the stepwise imputation was higher (0.65) than that of direct imputation from the BovineSNP50 beadchip to sequence data (0.37) or even than that of direct imputation from the BovineSNP50 beadchip in scenario S80 (0.46). Such a high accuracy with the stepwise approach was unexpected, because less information was available in the reference set. In the two-step approach, 20% of the individuals had genotypes similar to those of the BovineSNP50 panel (validation individuals), 40% had genotypes similar to those of the BovineHD panel (reference individuals step 1), and 40% had sequenced genotypes (reference individuals step 2). Whereas, in scenario S80, with direct imputation from the BovineSNP50 panel to sequence data, all reference individuals (80% of all individuals) had sequenced genotypes. VanRaden *et al.*[13] found an increase in imputation accuracy of about 2% when imputation was done from 3000 SNPs to 50 000 SNPs and then to 777 000 SNPs compared to direct imputation from 3000 SNPs to 777 000 SNPs. Although less information is used, the reason why there is this increase in imputation accuracy is not clear. However, one reason could be that the imputation algorithm has problems with selecting the correct haplotypes since there are multiple possible matches between sequence haplotypes and a BovineSNP50 haplotype, whereas there are less possible matches when BovineHD genotypes are added in between. In this case, there is a higher probability of selecting the long range haplotypes in the first step, and the short range haplotypes in the second step, which increases accuracy of imputation.

In cattle, many individuals with BovineHD genotypes are available. Using those individuals to impute BovineSNP50 genotypes to BovineHD genotypes may increase the accuracy gained in the first step, which would result in even higher accuracies when using the two-step approach than those obtained here. In some species, this is not a realistic scenario because no high-density marker panel is available yet, i.e. for pig. Developing these high-density panels and re-genotyping individuals can be expensive, especially if the end goal is to impute to sequence genotypes. In a scenario in which no high-density panel is available, it might be more cost effective to sequence additional animals and use the two-step approach by masking part of the SNPs of the individuals used for the first imputation step. This will mimic a high-density marker panel, and according to the results reported here, the overall imputation accuracy would be higher than that obtained by direct imputation from the lower density SNP chip. An improvement of this step-wise approach could be to use information of all individuals in the reference population in both steps instead of using disjoint reference sets as was done in this study, to mimic dairy cattle breeding practice. In the former case, the expected advantage is that all the genotype information will be available in the last step, while with disjoint datasets, the masked genotype information of individuals in the first step is not used in the second step. Moreover, it would be interesting to investigate the use of more than two steps because there may be an optimum number of steps to reach the highest accuracy.

In genomic selection, it is important to know the imputation accuracy per individual, because there is a direct relation with the accuracy of genomic prediction [34] and therefore the response of selection. In the present study, mean imputation accuracy per individual was higher compared to mean imputation accuracy per SNP, which was also reported by Mulder *et al.*[34]. They argued that allele frequencies bias imputation accuracy per individual and suggested to subtract mean genotype per SNP from observed and imputed genotypes. We tested this hypothesis and showed it had a small effect i.e. the mean accuracy of imputation from the BovineHD panel per individual in scenario S80 decreased only by 0.04 to reach 0.90. After standardization for the genotype variance per SNP, mean accuracy of imputation per individual in scenario S80 decreased furthermore to 0.87. This standardized mean accuracy per individual is still higher compared to the mean accuracy per SNP, however, the remaining bias is small and might be explained by a correlation between imputations of markers within a haplotype within an individual [34].

### Imputing SNPs with a low MAF

Using whole-genome sequence data for genomic prediction and GWAS is interesting because the actual polymorphisms that cause genetic differences are potentially included in the data e.g. [1-3]. The distribution of allele frequencies of causal mutations is not known, but it is hypothesized that those mutations may have a low MAF [1]. To calculate imputation accuracy, all SNPs with fixed observed genotypes or estimated *B*-allele dosages for one or more validation groups were removed. The remaining numbers of SNPs per scenario and per SNP chip are in Table 1. In the case of imputation from the BovineHD panel in scenario S80, 744 896 SNPs remained and 992 575 SNPs were removed from the dataset. It is possible that removing these SNPs without changing the allele dosage affected the results. Of the removed SNPs, 40.6% had a MAF of 0, which could have been easily imputed with a 100% accuracy, 56.1% had a MAF between 0 and 0.1 and their imputation accuracy could have been affected by their low MAF only, and the remaining 3.3% had a MAF above 0.1, which could have been difficult to impute for other reasons than their low MAF. However, it is unlikely that these 3.3% SNPs could affect the average imputation accuracy of common markers because of their small number. Although many loci with a low MAF in the observed genotypes were removed, among the remaining SNPs those with a lower MAF were more difficult to impute correctly and the reliability of imputation varied more than for the SNPs with a higher MAF. These findings may potentially limit the benefit of using imputed sequence data for genomic prediction and GWAS. However, decay in imputation reliability for SNPs with a lower MAF was smaller in the scenarios with more reference individuals than those with less reference individuals, which confirms results with human data [5]. In large-sized reference populations, there is more chance to have multiple allele copies to construct the haplotypes [16]. Moreover, Howie *et al.*[35] showed that a multi-population reference panel can improve imputation accuracy for SNPs with a low MAF, because a low-frequency allele in one population can be more frequent in another population. Since it is expected that, in the near future, more individuals from more different breeds will be sequenced in cattle, it is assumed that imputation accuracy of SNPs with a low MAF will improve.

Still, in species with a small number of sequenced individuals, imputation of SNPs with a low MAF may remain an issue. In such a situation, it might be beneficial to use another algorithm for imputation, such as IMPUTE [8] or MaCH [7]. It is claimed that these methods perform better compared to Beagle when the number of reference individuals is low [36,37] and for SNPs with a low MAF [38]. All three methods use Hidden Markov models, but IMPUTE and MaCH model genotypes on a set of haplotypes without clustering, whereas Beagle uses haplotype clustering strategies and therefore may miss SNPs with a low MAF [36,38]. Clustering strategies as in Beagle reduce computer time and memory use compared to IMPUTE and MaCH, which is an advantage when handling large datasets [37].

### Imputation reliability per SNP

Although the assumption that the polymorphisms responsible for genetic differences are included in the dataset may be true for sequence data, for imputed sequence data it is important to know if polymorphisms are imputed correctly. Beagle calculates an allelic R^2^ measure, which predicts accuracy of imputation per SNP. Allelic R^2^ is the squared correlation between allele dosage of the most likely imputed genotype and allele dosage of the true imputed genotype [5] and the closer these are, the more accurate the imputation is for the SNP. The correlation between the allelic R^2^ measure from Beagle and true imputation reliability that we calculated was equal to 0.79 for imputation from the BovineHD beadchip to sequence data in scenario S80 (results not shown). Of the 622 862 SNPs with estimates for both measures, 67,2% showed a difference between the allelic R^2^ measure from Beagle and true imputation reliability of less than 0.1, although the maximum difference between both measures was 0.78. This indicates that the allelic R^2^ measure provided by Beagle gives a good indication of imputation reliability in general, although in specific cases it may severely underestimate imputation reliability.

In human studies, imputed genotypes did not result in a high increase in power in GWAS compared to lower density marker panels [31,39,40]. Therefore, it is important to understand the underlying factors that affect imputation reliability and to take those factors into account when imputing genotypes. An important factor that influences imputation reliability is the LD between the imputed SNP and the SNP on the lower density marker panel. This may reduce the advantage of using imputed sequence data for genomic predictions or GWAS, compared to true sequence data. The advantage with true sequence data is the lack of dependency on LD between an SNP and the causal mutation in the sequence data, assuming that the true causal variant was accurately identified in the data. Our results showed that successful imputation of the causal mutation depended on the LD between the SNP on the lower density marker panel and the causal mutation. Hence, causal mutations that are poorly tagged by the low-density SNP panel will also be difficult to detect for reliable imputation.

In the current Holstein Friesian population, the effective population size is estimated to be around 100 [20,21]. However, Figure 4 shows that the decay in imputation accuracy based on a *Ne* of 1000 seemed more appropriate for our data than a *Ne* of 100. Hayes *et al*. [41] reported that LD at very short distances is related to effective population sizes in the past, while LD at longer distances is related to current effective population sizes. In our study, LD was calculated on very short distances, which suggests that a historical value should be used for *Ne*, rather than the current value of 100. Another reason for imputation reliability to decay more quickly than that expected from the decay in LD based on a *Ne* of 100 is that other factors also affected imputation reliability, or that the factors interacted with respect to their effect on accuracy. For example, when the SNP selected on the high-density panel and the SNP in the sequence are close, their MAF may be comparable, while as the distance between them increases the difference in MAF may also increase. Since these factors, distance and MAF, have a multiplicative effect, the decay in imputation reliability is larger than that expected from the decay in LD based on a *Ne* of 100. This expectation is confirmed by the resemblance between the combined functions for *Ne* of 100 (results not shown) and the combined functions for *Ne* of 1000 (Figure 7).

Another factor that affected LD was the difference in MAF, which at first sight may be an unexpected indicator for imputation accuracy, especially since haplotypes are used for imputation. However, as shown in other studies [25,28,29] the difference in MAF determines the mathematical upper limit of the LD between two SNPs. At extreme differences in MAF, alleles at the different SNPs cannot match, even if the distance between SNPs is small. For example, the maximum possible correlation obtained for two random binary variables with a MAF of 0.45 and 0.05, respectively, is 0.06. Thus, for two SNPs at the same distance, LD may differ and they may be in different haplotypes used for imputation. This could be particularly important since the SNPs included in the SNP panels are not randomly selected and generally have a high MAF.

Imputation reliability was also affected by the MAF of the imputed SNPs and by the number of sequenced individuals. Our results indicate that, if causal mutations have a low MAF, a large-sized reference group is required to impute those mutations correctly and to benefit from using sequence data, which confirms previous reports [1,42]. Extrapolation of *K*_*m*_ using a power function (R^2^ = 0.999) showed that, with more than 500 reference individuals, the increase in imputation reliability was expected to be small (results not shown). This agrees with other cattle studies that used lower density marker data and showed that, with more than 1000 reference individuals, the increase in imputation accuracy is expected to be small [12,32].

The goal of imputation is to assemble a large group of individuals with phenotypic information and sequence genotypes for genomic prediction or GWAS. For power calculations in GWAS, imputation reliability (not only overall imputation reliability but also imputation reliability per SNP because of the variation between SNPs) should be taken into account when imputed genotypes are used [8]. Our results show that functions that estimate LD based on distance only or on the difference in MAF between the imputed SNP and the closest SNP on the lower density marker panel did not provide a good indication of imputation reliability. When these functions were combined with an empirical derived function that corrects for MAF of the imputed SNPs and size of the reference group, a much better indication of imputation reliability was obtained but it was still not perfect (Figure 7). The same functions also held for BTA29, even when using estimates for *V*_*max*_ and *K*_*m*_ based on BTA1 (results not shown). Hence within this population and dataset, the predictions hold across chromosomes, at least on average since bins of 1000 SNPs were used. However, these functions could be further improved. For example, currently the functions are based on the use of an individual SNP (the closest SNP or the SNP in highest LD of the five closest SNPs) to estimate imputation reliability, whereas a program like Beagle uses haplotypes for imputation. Moreover, instead of choosing the closest SNP, a more distant SNP might be in higher LD with the imputed SNP. Therefore, using all SNPs or haplotypes is likely to estimate imputation reliability better than the functions used here. However, taking all SNPs into account or using haplotypes will make estimation more time-consuming and less generic applicable. Further research using simulation is necessary to investigate the generality of the estimations and the obtained imputation reliability. However, our study shows that the functions described above provide a good indication of the factors that affect imputation reliability per SNP.

Obviously, imputation reliability does not rely only on LD, MAF, and reference group size. Other factors, such as genotyping errors [36], or degree of relationship between validation and reference groups [9,10,32], are also important. It has been reported that increasing the number of close relatives in the reference group increased accuracy of imputation and that this increase was more pronounced when the differences between number of SNPs genotyped in the validation and reference populations were large (such as the differences between BovineSNP50 or BovineHD and sequence data) [10].

## Conclusions

Accuracy of imputation to whole-genome sequence data was generally high for imputation from the BovineHD beadchip, but was low for imputation from the BovineSNP50 beadchip. Stepwise imputation from the BovineSNP50 to the BovineHD beadchip and to sequence data substantially improved accuracy of imputation. SNPs with a lower MAF were more difficult to impute correctly and led to more variation in reliability of imputation. Functions that estimate LD based on distance only or on the difference in MAF between the imputed SNP and the closest SNP on the lower density marker panel did not provide a good indication of imputation reliability. However, when these functions were combined with an empirical derived function that corrects for MAF of the imputed SNPs and size of the reference group, estimation of imputation reliability was greatly improved.

## Competing interests

The authors declare that they have no competing interests.

## Authors’ contributions

RvB participated in the design of the study, performed the statistical analyses, and drafted the manuscript. MCAMB, MPLC, FAvE, and RFV participated in the design of the study and helped to draft the manuscript. BJH and IH contributed the genotype data. All authors read and approved the final manuscript.
